# Supplementary material for: Incidence of electrocardiographic and electrolyte changes in acute oleander poisoning in humans: A systematic review and meta-analysis protocol
Source: PLoS One. 2025 Mar 25;20(3):e0320301. doi: 10.1371/journal.pone.0320301 (PMC11936191; doi:10.1371/journal.pone.0320301)
Supplement: Attachment — Submitted filename: Review comments.docx [file pone.0320301.s002.docx]

Manuscript No.: **PONE-D-24-51489**

Manuscript Title: **Incidence of Electrocardiographic and Electrolyte Changes in Acute Oleander Poisoning in Humans: A Systematic Review and Meta-analysis Protocol**

The manuscript presents a systematic review and meta-analysis protocol focusing on the incidence of electrocardiographic (ECG) and electrolyte changes in patients with acute oleander poisoning. This is a timely and relevant topic given the significant health burden posed by oleander toxicity in many parts of the world. The methodology appears robust, adhering to established guidelines such as PRISMA-P and the JBI manual for evidence synthesis. Below are specific comments and suggestions for improvement.

Comments:

- The stated objectives should be specifying how the findings will directly impact clinical practice, particularly in resource-limited settings.
- The introduction section needs a brief discussion of existing gaps in the literature and how this review aims to address them.
- The introduction has more contexts on global incidence and relevance outside South Asia would enhance the generalizability of the review.
- The planned use of the JBI Critical Appraisal Tool is useful to specify how the findings from this tool will influence the synthesis and interpretation of the results.
- The discussion acknowledges limitations regarding heterogeneous data and missing information. However, strategies for mitigating these challenges (e.g., sensitivity analyses) should be expanded.
- Ethical approval is not required for this review, but the manuscript should emphasize plans for data sharing and transparency post-publication.
- To provide a rationale for excluding non-English studies and how this might impact results.
- Ensure consistency in referencing throughout the manuscript.
