# Supplementary material for: Incidence of electrocardiographic and electrolyte changes in acute oleander poisoning in humans: A systematic review and meta-analysis protocol
Source: PLoS One. 2025 Mar 25;20(3):e0320301. doi: 10.1371/journal.pone.0320301 (PMC11936191; doi:10.1371/journal.pone.0320301)
Supplement: Attachment — Submitted filename: Review.docx [file pone.0320301.s003.docx]

Comments to Editor

1. Language check with help of some software

2. Check spelling and grammatical mistakes throughout the manuscript.

3. Revise the whole manuscript as per the journal guidelines.

4. Latest references must be used.

5. In Introduction part Na+/K+ is change into Na^+^/K^+^.

6. Page No.4. Eligibility Criteria part – Verify the first line (according to CoCoPop).

7. Methodology should be precise and clear.
