# Supplementary material for: Incidence of electrocardiographic and electrolyte changes in acute oleander poisoning in humans: A systematic review and meta-analysis protocol
Source: PLoS One. 2025 Mar 25;20(3):e0320301. doi: 10.1371/journal.pone.0320301 (PMC11936191; doi:10.1371/journal.pone.0320301)
Supplement: Attachment — Submitted filename: Response to Reviewers.docx [file pone.0320301.s004.docx]

**2^nd^ February 2025**

Editor-in-Chief

PLOS ONE

**Subject: Rebuttal Letter for Manuscript ID PONE-D-24-51489**

Dear Editor,

We sincerely thank you and the reviewers for the constructive comments and suggestions provided for our manuscript titled Incidence of electrocardiographic and electrolyte changes in acute oleander poisoning in humans: A systematic review and meta-analysis protocol”. We have carefully reviewed all the feedback and made the necessary revisions to improve the quality of the manuscript. Below, we provide our point-by-point responses to the reviewers’ comments.

**Reviewer 01**

| Reviewer Comment | Revision | Line Number |
| --- | --- | --- |
| The stated objectives should be specifying how the findings will directly impact clinical practice, particularly in resource-limited settings | The objective was rephrased and explained how the objective will directly impact clinical practice in resource-limited settings | 115 -124 |
| The introduction section needs a brief discussion of existing gaps in the literature and how this review aims to address them. | A brief discussion of existing gaps in the literature is added to the introduction section while explaining how this review aims to address them. | 102 -113 |
| The introduction has more contexts on global incidence and relevance outside South Asia would enhance the generalizability of the review. | Global incidence was added and generalizability was enhanced | 55-62 |
| The planned use of the JBI Critical Appraisal Tool is useful to specify how the findings from this tool will influence the synthesis and interpretation of the results. | The use of the JBI critical appraisal tool in terms of the evidence synthesis and interpretation of the results was further explained. | 130-134 |
| The discussion acknowledges limitations regarding heterogeneous data and missing information. However, strategies for mitigating these challenges (e.g., sensitivity analyses) should be expanded. | Heterogeneous data and missing information as potential limitations were discussed along with the strategies of mitigation used discussion section. | 292-302 |
| Ethical approval is not required for this review, but the manuscript should emphasize plans for data sharing and transparency post-publication. | Plans for data sharing and transparency post-publication were added under the data accessibility section. | 275-280 |
| To provide a rationale for excluding non-English studies and how this might impact results. | The rationale for using only studies in English was explained and it was identified as a limitation of the systematic review. | 156-161 |
| Ensure consistency in referencing throughout the manuscript. | Added more references and ensured consistency of referencing throughout the protocol. | 320-399 |

**Reviewer 02**

| Reviewer Comment | Revision | Line Number |
| --- | --- | --- |
| Language check with the help of some software | Language check was done using Grammarly software |  |
| Check spelling and grammatical mistakes throughout the manuscript. | Language check was done using Grammarly software |  |
| Revise the whole manuscript as per the journal guidelines. | Necessary formatting and syles were done as per the POLS ONE style templates. |  |
| Latest references must be used.  The review needs more references regarding the study of oleader poisoning. The data provided is also insufficient. | New references with more research studies and more data were added and ensured consistency of referencing throughout the protocol. | 320-399 |
| In the Introduction part, Na+/K+ is changed into Na+/K+. | Nessasry correction was made | 65 |
| Page No.4. Eligibility Criteria part – Verify the first line (according to CoCoPop). | Verified the eligibility criteria and a reference was added. | 142-143 |
| The methodology should be precise and clear. | The methodology was re-arranged in a precise and clear manner | 125-280 |
| Have the authors described where all data underlying the findings will be made available when the study is complete? | A ststement was added under data accesasibility section to ensure all data underlying the findings described in their manuscript fully available without restriction | 275-280 |

In summary, we have incorporated all the reviewers’ suggestions to the best of our ability, and we believe the revised manuscript has been significantly improved as a result. A detailed list of changes is highlighted in the revised manuscript for your convenience.

We deeply value the opportunity to address these comments and appreciate your efforts in reviewing our work. Please feel free to contact us if further clarifications or revisions are needed.

Thank you for considering our revised manuscript for publication in PLOS ONE

Sincerely,

**Dr. Asanka Eriyawa**

**1st Author (Corresponding Author)**

**Prof. Pradeepa Jayawardane**

**(Corresponding Author)**
